# Staphylococcus Aureus Toxins and Asthma: Pathophysiological Mechanisms, Clinical Relevance, and Therapeutic Implications in the Biologics Era

**DOI:** 10.3390/toxins18080319

**Published:** 2026-07-23

**Authors:** Diego Bagnasco, Benedetta Bondi, Greta Losacco, Carola Montagnino, Francesca Froio, Elena Tedesco, Gloria D’Alessandro, Ilaria Baglivo, Laura Bruno, Sara Chiappori, Maria José Murillo Jaramillo, Marcello Mincarini, Fulvio Braido, Cristiano Caruso

**Affiliations:** 1 Respiratory and Allergy Clinic, IRCCS Azienda Ospedaliera Metropolitana (AOM), Plesso: Ospedale Policlinico San Martino, Largo Rosanna Benzi, 16132 Genoa, Italy; 2 Department of Internal Medicine (DIMI), University of Genoa, Viale Benedetto XV, 16132 Genoa, Italy; 3 Department of Translational Medicine and Surgery, Università Cattolica del Sacro Cuore, Largo Francesco Vito, 00168 Rome, Italycristiano.caruso@policlinicogemelli.it (C.C.); 4 UOSD Allergology and Clinical Immunology, Fondazione Policlinico Universitario Agostino Gemelli IRCCS, 00168 Rome, Italy

**Keywords:** *Staphylococcus aureus*, staphylococcal enterotoxins, superantigens, severe asthma, IgE, eosinophils, chronic rhinosinusitis with nasal polyps, epithelial barrier, biologics, microbiota, atopic dermatitis, extracellular vesicles, biofilm, colonization

## Abstract

Staphylococcus aureus frequently colonizes the skin and upper airways and produces a broad repertoire of immunomodulatory molecules. In asthma, the most consistent evidence concerns staphylococcal enterotoxins (SEs), which can act both as superantigens and as allergens, and IgE sensitization to SEs (SE-sIgE). SE-sIgE is associated with severe asthma, type 2 inflammation, chronic rhinosinusitis with nasal polyps (CRSwNP), exacerbations, and, in some longitudinal studies, persistent airflow obstruction. However, this relationship is not necessarily causal: SE-sIgE may reflect exposure, an immune response, or a biologically active endotype, whereas colonization, local toxin production, and systemic sensitization are not equivalent. SEs simultaneously bind class II MHC molecules and Vbeta regions of the T-cell receptor, activating large fractions of T lymphocytes; they also promote IL-4, IL-5, and IL-13 production, polyclonal B-cell activation, local IgE synthesis, mast-cell degranulation, eosinophilia, and IL-8/neutrophil circuits. Alpha-toxin (Hla) and SEB can damage the epithelial barrier, facilitating allergen penetration and alarmin signalling. These observations support an interaction model in which dysbiosis, barrier dysfunction, and type 2 immunity mutually reinforce one another along the nasobronchial axis. Corticosteroids and antibiotics may modify selected nodes in this circuit, but current evidence is insufficient to recommend decolonization or antitoxin therapy in stable asthma. Biologics interrupt downstream pathways potentially fuelled by toxins: omalizumab neutralizes free IgE; mepolizumab and benralizumab reduce the eosinophilic axis; dupilumab blocks IL-4/IL-13 signalling; and tezepelumab acts upstream on TSLP. Nevertheless, randomized trials stratified by SE-sIgE are lacking, and no evidence demonstrates that these treatments eliminate colonization or toxin production. SE-sIgE therefore appears to be a promising biomarker, particularly in severe asthma with CRSwNP, but it is not yet an autonomous criterion for biologic selection. A broader barrier-organ analysis also identifies nasal, cutaneous, and intestinal colonization as distinct ecological states; atopic dermatitis as a complementary model of toxin-amplified type 2 inflammation; and biofilms and extracellular vesicles as candidate mechanisms of persistent toxin delivery. These data increase biological plausibility but remain indirect for asthma.

## 1. Introduction

Asthma is a heterogeneous inflammatory syndrome, defined by respiratory symptoms and variable airflow limitation, in which similar clinical phenotypes may arise from different endopathophysiological pathways. In severe asthma, type 2 (T2) inflammation remains dominant in a substantial proportion of patients. Still, infections, dysbiosis, and products derived from host-microbe interactions may amplify or redirect immune responses and contribute to disease persistence [1,2].

*S. aureus* is simultaneously a commensal, a reservoir organism, and an opportunistic pathogen. Its biological effect depends on anatomical niche, host barrier integrity, strain-specific virulence, and the local immune environment. Surface adhesins, immune-evasion proteins, secreted toxins, extracellular enzymes, and biofilm formation allow the organism to persist across mucosal and cutaneous sites and to shift from asymptomatic carriage to tissue injury [3,4,5].

Among microorganisms that may modify asthma through airway colonization, epithelial injury, and immune activation, Staphylococcus aureus is of particular interest. This bacterium can stably colonize several tissues, including the nasal vestibule, paranasal sinus mucosa, and skin, without causing invasive infection; under favorable conditions, it produces several proteins, including staphylococcal enterotoxins (SEs), toxic shock syndrome toxin-1 (TSST-1), alpha-hemolysin/alpha-toxin (Hla), leukocidins, exfoliative toxins, and protein A [6,7,8]. In the asthma literature, the term “enterotoxin sensitization” generally refers to serum IgE against SEA, SEB, SEC, or a mixture containing SEA/SEC/TSST-1. Importantly, their detection does not directly document either current colonization or toxin production in the airways.

An early meta-analysis estimated an association between SE-sIgE and asthma (pooled odds ratio 2.95), although with marked heterogeneity [9]. Subsequent studies also linked SE-sIgE to severe asthma, late onset, eosinophilia, and CRSwNP [10,11]. The Italian multicenter study by Caruso et al., which included 249 patients with severe asthma, detected SEB-sIgE in 25.3% of patients and found an association with chronic rhinosinusitis and nasal polyposis, but not with conventional atopy, exacerbations, or corticosteroid dose [12]. This apparent discordance is relevant: SEs may be better interpreted as contextual disease modifiers rather than universal determinants of severity.

The present review therefore places the airway evidence within a broader barrier-organ framework. Data from intestinal carriage and AD are included when they clarify colonization ecology, epithelial injury, toxin delivery, or type 2 amplification. They are not treated as direct evidence of bronchial causality.

## 2. *S. aureus* Colonization, Persistence, and Transition to Disease

### 2.1. Colonization Across Anatomical Niches

Colonization is the first step in the *S. aureus*–host relationship and should be distinguished from infection and toxin-mediated inflammation. The anterior nares are the best-characterized reservoir, but the pharynx, skin, and gastrointestinal tract may also sustain carriage. Persistent and intermittent carriage reflect differences in host immunity, microbial competition, epithelial receptors, and bacterial adaptation; carriage at one site does not guarantee carriage at another [4,5,13,14].

In the nose, wall teichoic acids and surface adhesins promote attachment to epithelial and extracellular-matrix components. On skin, proteins including clumping factor B and SasG facilitate adherence, while the intestinal niche is influenced by mucus, diet, antibiotic exposure, and competition from resident microorganisms [3,13,15]. These ecological distinctions are clinically important: a positive nasal culture cannot be used as a surrogate for bronchial colonization, active toxin expression, or systemic sensitization.

### 2.2. From Colonization to Invasion and Immune Evasion

Barrier disruption changes the relationship from coexistence toward inflammation or invasion. Epithelial sensing, including Toll-like receptor 2 signalling, induces cytokines, chemokines, and antimicrobial peptides; resident mast cells and macrophages recruit neutrophils, natural killer cells, and IL-17-producing lymphocytes. *S. aureus* counters these defences through antiphagocytic and complement-evasion mechanisms, interference with neutrophil killing, intracellular persistence, and impairment of durable adaptive immunity [16,17,18,19].

Virulence is dynamically regulated rather than constitutively expressed. Quorum sensing and global regulatory networks integrate bacterial density with environmental and host signals, shifting production between surface-associated colonization factors and secreted enzymes or toxins. Mobile genetic elements and strain-level genomic variation further generate heterogeneous virulence and antimicrobial-resistance profiles [5,19]. This explains why detection of the organism, a toxin gene, toxin protein, and toxin-specific IgE represent biologically different measurements. In *S. aureus*, the accessory gene regulator (agr) quorum-sensing system, together with regulators such as SaeRS, SarA, and sigma B, responds to bacterial density, oxygen tension, pH, nutrient limitation, antimicrobial peptides, and epithelial or inflammatory stress; these signals determine whether adhesins, immune-evasion proteins, enzymes, or toxins predominate.

### 2.3. Intestinal Carriage and the Gut–Airway Hypothesis

*S. aureus* can colonize the intestine even in the absence of nasal carriage, although nasal carriers have a greater probability of concurrent gastrointestinal colonization. Intestinal carriage is more frequent in hospitalized, antibiotic-exposed, or vulnerable populations and may act as a reservoir for recolonization or invasive infection [14,15]. Antibiotic-associated dysbiosis reduces colonization resistance, while disruption of mucus and epithelial tight junctions may facilitate bacterial or product translocation.

The mature gut microbiota normally provides ecological resistance through diversity, metabolic competition, and inhibitory metabolites. Reported microbiota signatures of methicillin-sensitive and methicillin-resistant *S. aureus* carriage remain inconsistent, and causal direction is uncertain [15,20]. A gut–airway connection through microbial metabolites, circulating immune cells, or systemic barrier signals is plausible, but no study has established that intestinal carriage predicts airway toxin levels, SE-sIgE, asthma severity, or response to biologics. For this reason, response to biological therapeutics should be interpreted as downstream immune modulation, not as proof that *S. aureus* colonization or toxin production has changed.

## 3. Toxin Repertoire and Relevance to the Airways

Classical SEs (SEA-SEE) more recently described enterotoxins, and TSST-1 belong to the superantigen family. Their functional hallmark is cross-linking between MHC-II on antigen-presenting cells and specific variable beta (Vbeta) families of the TCR, outside the conventional peptide-binding groove. This enables activation of a much larger fraction of T lymphocytes than an ordinary antigen, with release of IL-2, IFN-gamma, TNF, and, in an atopic microenvironment, interleukin (IL)-4, IL-5, and IL-13 [6,21]. At chronic mucosal doses, the result is less like systemic toxic shock and more consistent with persistent disruption of local homeostasis. Unlike conventional peptide antigens, SEs do not require intracellular processing or peptide-specific recognition; by cross-linking MHC-II and selected TCR-Vbeta families outside the peptide groove, they can activate a broad, oligoclonal T-cell pool and secondarily promote polyclonal B-cell activation and IgE class switching.

Hla, by contrast, is a pore-forming toxin. The monomer binds to the membrane, with ADAM10 serving as an important functional receptor in several cell types, oligomerizes into a beta-barrel pore, and alters ion fluxes, the cytoskeleton, and junctional integrity. In respiratory epithelial cells, Hla induces loss of actin fibers, cofilin activation, ATP release, and the appearance of paracellular gaps [22,23,24]. Bicompartment leukocidins primarily target leukocytes; their direct role in asthma has not been demonstrated, but they may modify responses to colonization or infection. Protein A and lipoteichoic acid are not toxins in the strict sense, but they cooperate with SEs in shaping mucosal responses [25].

Beyond soluble toxins, *S. aureus* releases extracellular vesicles (EVs), membrane-enclosed particles that can carry proteins, nucleic acids, and cytotoxic cargo. Experimental work shows that EVs can deliver alpha-toxin into epithelial cells and induce inflammatory injury; packaging may protect toxins from proteolysis or antibody neutralization and extend their effective range [26,27,28]. Direct evidence for EV-associated toxin delivery in human asthma is currently absent (Table 1).

Delta-toxin can promote mast-cell degranulation, while V8 protease and exfoliative toxins disrupt epithelial adhesion; exfoliative toxins A and B target desmoglein 1 in skin disease [29,30]. Biofilms add a separate persistence mechanism by shielding bacterial communities from immune clearance and antimicrobial exposure and by sustaining local release of soluble products [31,32]. These mechanisms are well described in cutaneous disease but require airway-specific validation.

**Table 1:** Main *S. aureus* products potentially relevant to asthma.

Product	Main Mechanism	Plausible Airway Effect	Level of Evidence in Asthma
SEA, SEB, SEC, and other SEs	Superantigens; MHC-II/TCR-Vbeta cross-linking; allergens capable of inducing sIgE	Polyclonal T-cell expansion, IL-4/IL-5/IL-13, local and systemic IgE, eosinophilia; possible IL-8/neutrophilia	Repeated clinical association for SE-sIgE; direct causality not demonstrated [6,21,25,33,34].
TSST-1	Superantigen; massive T-cell activation	Cytokine amplification; included in some diagnostic mixtures	Specific evidence is limited and often not separable from SEA/SEC [6,21].
Hla/alpha-toxin	ADAM10 binding, oligomerization, and pore formation	Barrier injury, actin rearrangement, ATP loss, inflammation	Robust cellular evidence; few direct data in human asthma [22,23,24].
Leukocidins (PVL, LukED, gamma-hemolysins)	Bicomponent pores on leukocytes	Cytolysis and remodeling of the innate response during infection	Indirect evidence; no validated asthma biomarker [7,8].
Protein A	Binding to Ig Fc regions and VH3 regions of BCRs; immune activation	Altered B-cell response and cooperation with other bacterial products	Mainly sinonasal/in vitro data [25].
Exfoliative toxins and phenol-soluble modulins	Proteolysis or membrane damage	Potential epithelial inflammation	Role in asthma not defined [29,30].
Delta-toxin	Mast-cell activation and degranulation	Potential amplification of immediate allergic inflammation	Cutaneous/experimental evidence; asthma role undefined [29].
Secreted proteases and exfoliative toxins	Cleavage of epithelial adhesion proteins, including desmoglein 1	Barrier disruption and increased access of allergens or toxins	Strong skin-disease evidence; limited airway evidence [31,32].
Toxin-containing extracellular vesicles	Protected transport and intracellular delivery of alpha-toxin and other cargo	Potential persistence and extension of toxin-mediated epithelial injury	Experimental/AD evidence; not demonstrated in asthma [26,27,28].
Biofilm-associated products	Persistent bacterial community with reduced immune and antimicrobial susceptibility	Sustained local exposure and recolonization	Plausible but not quantified in asthma [7,8].

## 4. Conceptual Framework

On the basis of this toxin repertoire, the following model considers *S. aureus* products as potential amplifiers of airway disease rather than as stand-alone causes of asthma. The framework distinguishes colonization, toxin expression, IgE sensitization, and downstream inflammation, because these layers may coexist but are not interchangeable in clinical interpretation. This distinction is especially important for asthma onset versus exacerbation: current evidence better supports *S. aureus* toxins as amplifiers of established type 2 airway disease or comorbid CRSwNP than as independent initiators of asthma in most colonized individuals.

## 5. Pathophysiological Mechanisms of Interaction with Asthma

### 5.1. Epithelial Barrier Injury and the Amplification Loop

The epithelium of patients with asthma often shows vulnerable tight and adherens junctions, impaired repair, and greater release of TSLP, IL-33, and IL-25 in response to stress. Hla may aggravate this vulnerability by forming pores and activating ADAM10; the resulting E-cadherin cleavage, actin disorganization, and loss of integrity increase paracellular passage [22,23,24]. SEB reduces transepithelial resistance and alters junctional proteins in nasal epithelial cultures; the reported effect includes oxidative and endoplasmic-reticulum stress [35].

**Figure 1:**
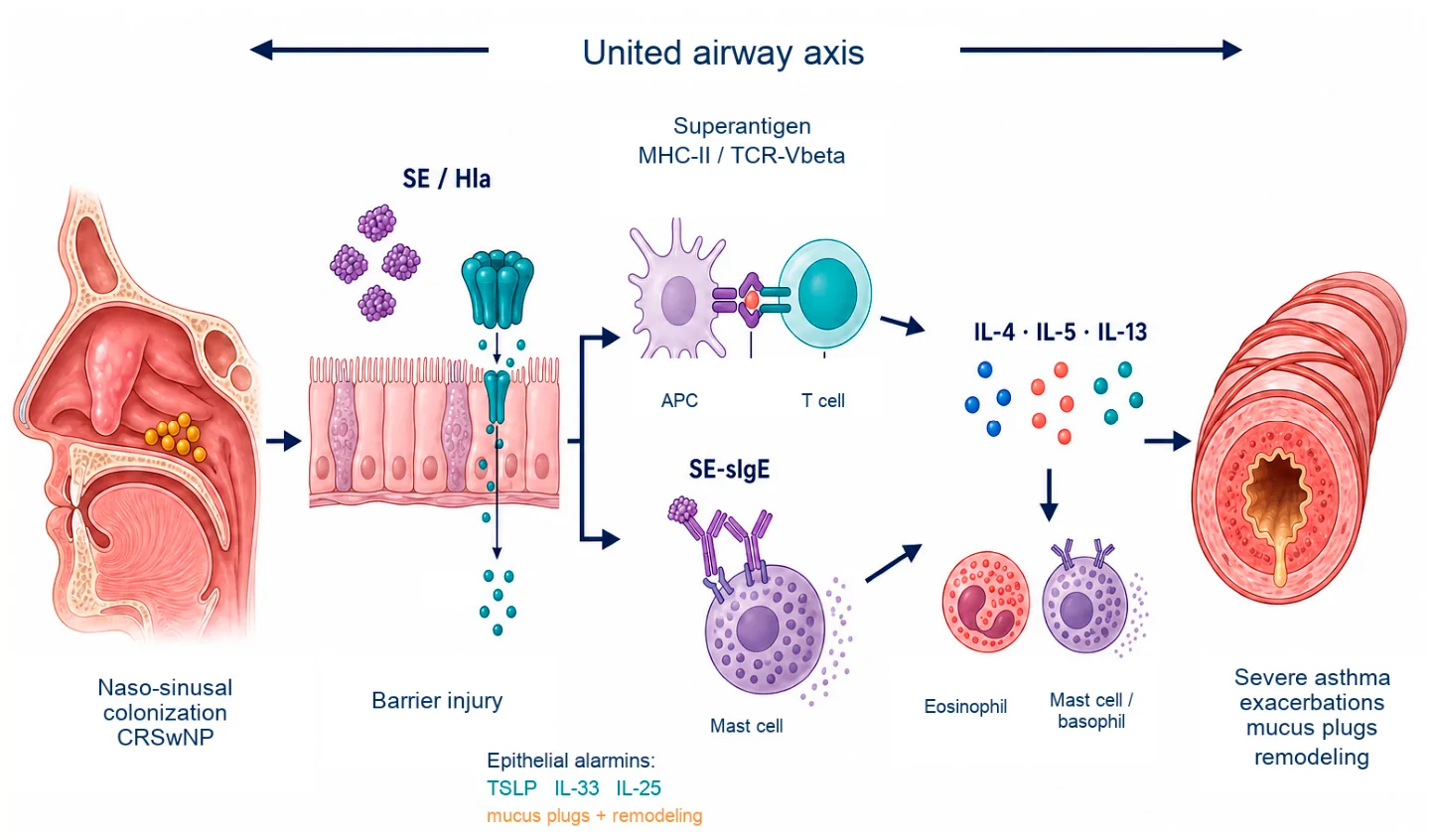
Integrated model of the interaction between Staphylococcus aureus toxins and asthma. Naso-sinusal colonization, particularly in CRSwNP, may expose the epithelium to staphylococcal enterotoxins (SEs) and alpha-toxin (Hla). Barrier injury promotes epithelial alarmins, including TSLP, IL-33, and IL-25, which may contribute to mucus plugs and airway remodeling. SEs act both as MHC-II/TCR-Vbeta superantigens and as allergens recognized by SE-sIgE. Convergence on IL-4, IL-5, and IL-13 sustains mast cells, eosinophils, exacerbations, and remodeling.

A damaged barrier simultaneously facilitates allergen and toxin access to the lamina propria, exposes immune cells to microbial signals, promotes ATP and alarmin release, and reduces mucociliary clearance. A bidirectional loop is therefore created. T2 inflammation weakens the barrier through IL-4 and IL-13, whereas a permeable barrier sustains microbial exposure. (Figure 1) Future mechanistic studies should therefore measure viral infection, particularly rhinovirus, at the same visits as colonization, toxin expression, SE-sIgE, and epithelial cytokines, because viral epithelial injury may independently trigger exacerbations and confound attribution to *S. aureus*.

### 5.2. Superantigenicity and T2 Polarization

SEs do not require canonical antigen processing. By binding MHC-II and TCR-Vbeta, they induce oligoclonal/polyclonal expansion, followed by a complex phase of activation, anergy, or cell death. In inflamed respiratory tissue, dendritic cells, macrophages, and B lymphocytes present SEs and release mediators that favor Th2 and ILC2 responses. IL-4 and IL-13 promote class switching toward IgE, mucus hypersecretion, and airway hyperresponsiveness; IL-5 sustains eosinophil maturation, recruitment, and survival [6,21,33].

This mechanism explains why SE-sIgE may also appear in patients defined as “non-atopic” on the basis of common inhalant allergens. SEs may simultaneously generate specific IgE and a polyclonal increase in total IgE. Activation is not confined to T lymphocytes: mast cells sensitized with SE-sIgE can degranulate when they encounter the toxin, while eosinophils and basophils amplify mediator release.

### 5.3. Local IgE Production and B-Cell Memory

CRSwNP provides the most convincing human model of *S. aureus*-driven local IgE production. Polyp tissue contains total and SE-specific IgE, plasma cells, and class-switch recombination signals. In 2024, Du and colleagues showed that *S. aureus* lysate induced IgE production in polyp cultures but not in healthy mucosa; single-cell RNA sequencing detected increased IL-5/IL-13 in ILC2s and clonal overlap between memory B cells and induced plasma cells [36]. These findings support a local memory B-cell/plasma-cell circuit, although they do not attribute the effect to a single toxin. However, comparable paired airway, blood, skin, and sinonasal datasets with TCR/BCR repertoire analysis are scarce, so current serum SE-sIgE cannot determine whether sensitization originated from respiratory exposure, sinonasal disease, skin disease, or multiple barrier sites.

Serum IgE against SEs is therefore an accessible window into a potentially compartmentalized process, but its sensitivity and specificity as a clinical biomarker are imperfect and should not be confused with the biological activity of SE-sIgE itself. A threshold of 0.35 kUA/L increases specificity, whereas values between 0.10 and 0.35 kUA/L may have epidemiological relevance. Different assays (single SEB versus SEA/SEC/TSST-1 mixtures), seasonality of colonization, and biologic treatment hinder direct comparisons.

### 5.4. Eosinophils, Mast Cells, and Neutrophils

The association between SE-sIgE, eosinophilia, and CRSwNP has been reproduced in several cohorts [10,11,12]. Activated eosinophils produce cationic proteins, leukotrienes, and extracellular traps; the latter have been observed together with *S. aureus* at epithelial defects in patients with severe airway inflammation [37]. The relationship may be reciprocal: traps contain the bacterium but increase viscosity, tissue damage, and antigen persistence.

SEs can also induce epithelial IL-8/CXCL8 release, favoring neutrophilia [34]. A prospective omalizumab abstract presented at the European Respiratory Society Congress 2023 suggested that SE-sIgE-positive patients had higher bronchial IL-8 and neutrophils, with a less complete clinical response; because these are conference data, they should not guide practice [38]. This mixed T2/neutrophilic component could nevertheless explain why neutralization of a single pathway may leave residual disease.

### 5.5. United Airway Axis: CRSwNP, N-ERD, and Asthma

The nasal cavity and paranasal sinuses constitute a plausible reservoir of *S. aureus* and soluble products. Eosinophilic CRSwNP, adult-onset asthma, and NSAID-exacerbated respiratory disease (N-ERD) share IL-4/IL-5/IL-13, eosinophils, mast cells, cysteinyl leukotrienes, and barrier dysfunction. SEs may overlap with this network by increasing local IgE and T-cell activation without requiring aeroallergen sensitization [12,39].

However, the coexistence of polyposis and SE-sIgE does not prove that the toxin “descends” into the bronchi. At least four models are possible: parallel nasal and bronchial exposure; spillover of systemic mediators; aspiration of secretions; or SE-sIgE as a marker of a shared immunoepithelial defect. Studies with simultaneous sampling, quantitative culture, strain-level metagenomics, and direct toxin measurement are needed to distinguish among these models.

### 5.6. Remodeling, Fixed Obstruction, and Corticosteroid Resistance

Repeated exposure to epithelial injury, eosinophils, and growth factors may favor mucous metaplasia, basement-membrane thickening, and smooth-muscle changes. In a prospective Korean cohort of 223 older adults with asthma, baseline SE-sIgE sensitization was associated with persistent obstruction after two years, and SE-sIgE levels correlated with exacerbations [40]. The longitudinal design strengthens temporality, but smoking, longer asthma duration, CRS, and immunosenescence remain potential confounders.

SEB also reduced glucocorticoid responsiveness in ex vivo models through intense immune activation and alterations in glucocorticoid-receptor signaling [41]. This provides a biological explanation for the difficult control of some phenotypes, but it does not prove that SE-sIgE predicts clinical steroid resistance. In a small 2024 cohort, 12 of 50 patients with severe asthma were SE-sIgE positive and showed worse FEV1 and FEV1/FVC, without consistent differences in histological remodeling indices [42]. Optimized treatment, often with biologics, may attenuate associations present in the natural history of the disease.

### 5.7. Atopic Dermatitis as a Complementary Barrier–Toxin Model

AD is a chronic inflammatory barrier disease and an important clinical analogue for the interaction among type 2 immunity, epithelial dysfunction, dysbiosis, and *S. aureus*. It often precedes asthma and allergic rhinitis within the atopic march, although progression is neither universal nor explained by a single microorganism [43,44]. Genetic and acquired reductions in filaggrin, increased transepidermal water loss, altered skin pH, and reduced antimicrobial defenses create a niche favorable to *S. aureus* expansion.

Skin microbiome studies consistently show reduced microbial diversity during AD flares and marked enrichment of *S. aureus*. A meta-analysis found carriage in approximately 70% of lesional skin and 39% of non-lesional skin, substantially above healthy controls; bacterial density correlates with disease severity [29,45,46]. Colonization and inflammation form a bidirectional loop: barrier dysfunction favors adherence and overgrowth, whereas bacterial toxins and proteases further damage the barrier.

Alpha-toxin is particularly relevant to this loop. Type 2 cytokines increase keratinocyte susceptibility to alpha-toxin-mediated death, and filaggrin-dependent sphingomyelinase activity provides a protective mechanism that is weakened when filaggrin expression is reduced [47,48]. Delta-toxin promotes mast-cell activation, whereas exfoliative toxins and proteases disrupt intercellular adhesion. These findings support the general concept that a type 2 microenvironment can magnify toxin injury, but the receptor distribution and exposure conditions of bronchial epithelium differ from skin.

More than one pathway links staphylococcal superantigens to AD. SEB and TSST-1 act as allergens capable of inducing specific IgE and mast-cell or basophil activation, while their superantigenic activity drives broad T-cell proliferation and cytokine release. SEB has also been linked to IL-31 expression, impaired keratinocyte differentiation, reduced filaggrin, and in vitro corticosteroid resistance [29,49,50]. These parallels strengthen the mechanistic plausibility of SE-mediated airway amplification without proving that skin sensitization and bronchial exposure are interchangeable.

EVs and biofilms may prolong this vicious cycle. *S. aureus* EVs containing alpha-toxin induce AD-like inflammation in experimental systems and can deliver cytotoxic cargo into keratinocytes [26,27,28]. Biofilm-producing staphylococci are common in AD lesions and may facilitate persistence despite immune pressure or treatment [31,32]. Measuring these delivery states in nasal polyps and lower-airway samples is a priority because conventional culture does not quantify active toxin, EV cargo, or biofilm-associated exposure.

**Figure 2:**
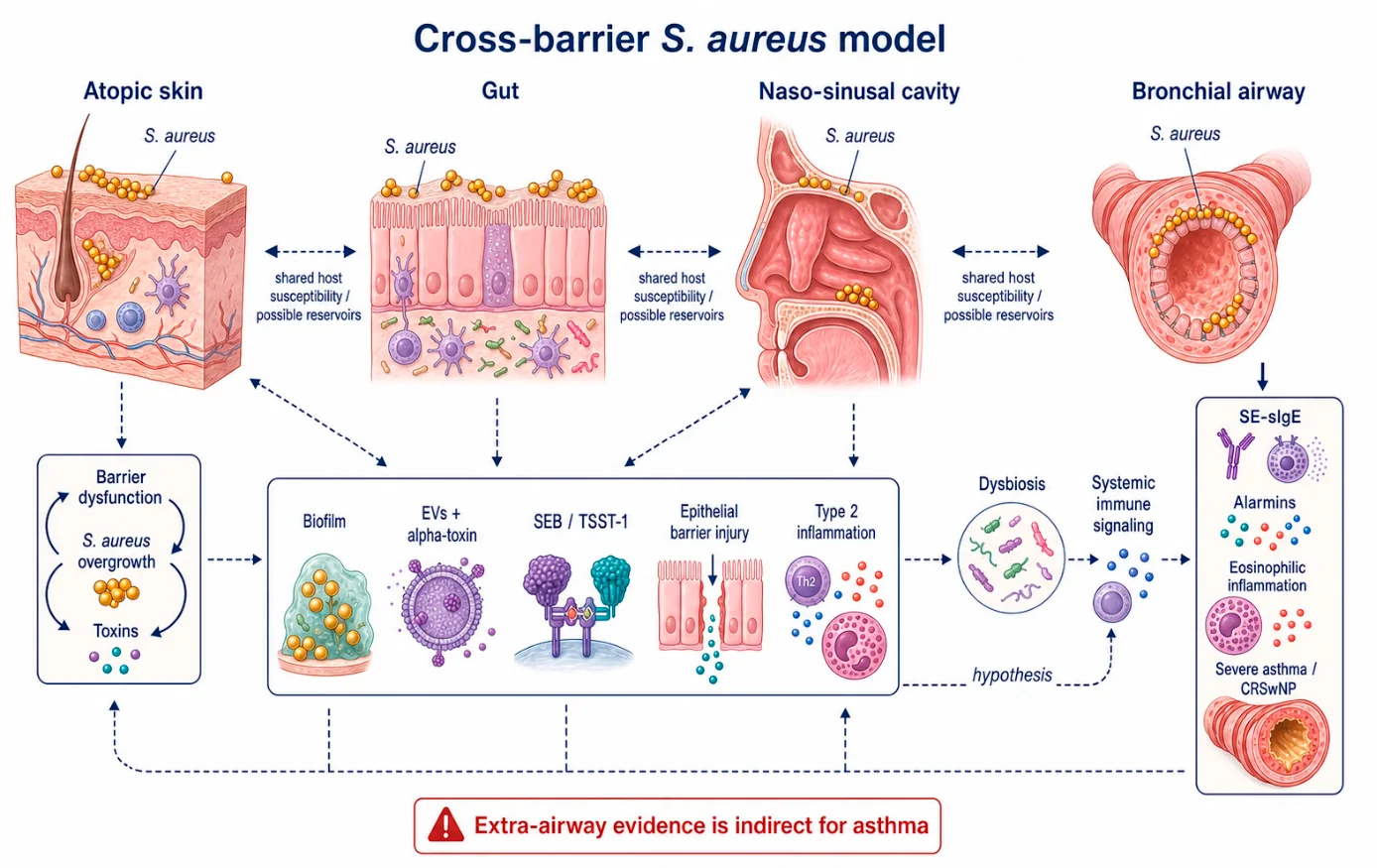
Cross-barrier model of Staphylococcus aureus colonization and toxin-mediated inflammation. Atopic skin, the gastrointestinal tract, the naso-sinusal cavity, and the bronchial airway represent distinct ecological compartments that may share host susceptibility and potential reservoirs. Biofilms, extracellular vesicles (EVs) carrying alpha-toxin, and superantigens such as SEB and TSST-1 may sustain epithelial injury and type 2 inflammation. In the gut, dysbiosis and reduced colonization resistance may influence systemic immune signaling; this gut-airway connection remains hypothetical. In the respiratory compartment, SE-sIgE, epithelial alarmins, and eosinophilic inflammation converge on severe asthma and CRSwNP. Evidence derived from extra-airway compartments remains indirect for asthma and does not establish inter-organ bacterial or toxin transfer.

Overall, the cross-barrier model of Staphylococcus aureus colonization and toxin-mediated inflammation, integrating the mechanisms described above, is summarized in Figure 2.

## 6. Clinical Evidence: What Does SE-sIgE Measure?

The literature converges on three observations. First, SE-sIgE is more frequent in asthma than in controls and increases with age and severity [9,10,11]. Second, the signal is particularly strong in adult/older, eosinophilic phenotypes associated with CRS/CRSwNP [10,12,40]. Third, associations are not uniform: in the Italian cohort by Caruso et al., SEB-sIgE did not correlate with exacerbations or corticosteroid dose [12], whereas the Korean cohort showed relationships with exacerbations and fixed obstruction [40].

These differences may reflect population, age, ethnicity, severity definition, the single toxin measured, analytical threshold, biologic use, and prevalence of polyposis. SE-sIgE should therefore be interpreted as a composite biomarker of exposure and immune response, not as the equivalent of a positive culture. Similarly, a positive nasal culture does not indicate the presence of a toxin gene, its expression, or the amount of biologically active toxin. The main clinical studies investigating the role of staphylococcal enterotoxin B (SEB) in asthma are summarized in Table 2.

Overall, the incremental clinical utility of SE-sIgE beyond eosinophils, FeNO, total IgE, age at onset, exacerbations, and CRSwNP has not been demonstrated. Before SE-sIgE can be incorporated into a decision algorithm, harmonized thresholds, longitudinal repeatability, and treatment studies with prespecified biomarker-treatment interaction are required.

## 7. Potential Effects of Therapies

### 7.1. Corticosteroids, Macrolides, and Comorbidity Treatment

Inhaled corticosteroids remain the cornerstone of persistent asthma and reduce numerous mediators downstream of SEs. However, SEB may generate relatively steroid-insensitive signals in vitro [41]. This does not justify indiscriminate dose escalation: cumulative exposure to systemic corticosteroids carries substantial toxicity, whereas biologics often allow meaningful steroid sparing [1,52].

Macrolides may reduce exacerbations in subgroups of persistent asthma through antibacterial and immunomodulatory effects, but they are not selective therapies against SEs. Resistance, QT prolongation, hearing effects, and nontuberculous mycobacteria should be considered before their use [53]. Saline irrigation, intranasal corticosteroids, appropriate surgery, and biologics for CRSwNP may reduce inflammation and secretion burden in the upper airway compartment, but they have not been shown to abolish toxin production.

### 7.2. Antibiotics and Decolonization

Isolated SE-sIgE positivity is not an indication for antibiotics. No adequate trials demonstrate that mupirocin, chlorhexidine, or systemic antibiotics stably improve asthma through eradication of *S. aureus*. Decolonization may be appropriate for recurrent infections or specific infectious-disease pathways, but not as standard asthma treatment. Biofilm formation, recolonization, and selection of resistance further limit the plausibility of durable benefit. The limited value of non-stratified antibiotic or decolonization approaches does not by itself refute a toxin-amplification model; rather, it indicates that future trials must enroll patients with documented carriage, active toxin production or SE-sIgE, and airway-specific inflammatory endpoints.

Antitoxin strategies, including Hla-neutralizing antibodies, pore-formation inhibitors, multicomponent vaccines, or quorum-sensing interference, are biologically interesting but remain preclinical or have been developed for invasive infections rather than asthma [54]. For a chronic disease, it would be necessary to demonstrate a therapeutic window capable of neutralizing low-level mucosal exposure without perturbing the microbiota.

Microbiome-directed interventions attempt to restore colonization resistance rather than eradicate *S. aureus* with conventional antibiotics. In a phase 2 randomized trial, oral Bacillus subtilis markedly reduced intestinal and nasal carriage without major disruption of the resident microbiota, supporting quorum-sensing interference as a potential non-antibiotic strategy [55]. Fecal microbiota transplantation has also restored diversity in small reports of MRSA enterocolitis [56]. Neither approach has been tested as an asthma treatment, and carriage reduction at a distant site cannot be assumed to reduce airway toxins or exacerbations.

### 7.3. Omalizumab: Neutralizing IgE, Not the Source

Omalizumab binds free IgE, reduces Fc-epsilon-RI on mast cells and basophils, and attenuates immediate and late responses. In theory, it neutralizes both IgE against aeroallergens and SE-sIgE, thereby potentially interrupting the allergenic arm of enterotoxins. However, it does not prevent MHC-II/TCR superantigen cross-linking, bacterial toxin production, or Hla-mediated injury.

The efficacy of omalizumab in severe allergic asthma and CRSwNP is well documented [2,57]. However, published trials demonstrating a differential benefit in SE-sIgE-positive patients are lacking. Conference data suggesting an attenuated response in the presence of IL-8/neutrophilia are hypothesis-generating [38]. Consequently, SE-sIgE is not currently a validated criterion for preferring or avoiding omalizumab.

### 7.4. Anti-IL-5 and Anti-IL-5Ralpha: Interrupting the Eosinophilic Effector Arm

Mepolizumab and reslizumab neutralize IL-5; benralizumab binds IL-5Ralpha and induces eosinophil depletion through antibody-dependent cell-mediated cytotoxicity. Because SEs may feed IL-5 production through Th2 cells and ILC2s, these drugs act on an important downstream effector, independently of IgE specificity.

Italian real-life data provide useful clinical context, although they were not designed as toxin-focused studies. Bagnasco, Caruso, and colleagues documented a sustained three-year reduction in exacerbations and corticosteroid use with mepolizumab, with oral steroid discontinuation in 87% of patients still under observation [58]. Pini and colleagues showed persistence of benralizumab effectiveness at three years [59]. A subsequent analysis from the same network found a reduction in inhaled corticosteroid dose in approximately 30% of patients during three years of benralizumab, whereas about two thirds remained on a stable dose [60]. These three contributions, together with the enterotoxin study [12] and the subsequent dupilumab-switch study discussed below, constitute the five Caruso and Bagnasco studies selected for this review. They support efficacy on T2 pathways, not a specific antistaphylococcal action.

CRSwNP may guide treatment choice: mepolizumab reduced the need for surgery and symptoms in the SYNAPSE study [61], whereas benralizumab improved endoscopic score and obstruction in the OSTRO study, with more variable effects on other outcomes [62]. A favorable united-airway response could indirectly reduce inflammatory niches favorable to *S. aureus*, but this hypothesis requires longitudinal cultures and toxin measurements.

### 7.5. Dupilumab: IL-4/IL-13, Barrier Function, and IgE Production

Dupilumab blocks IL-4Ralpha, which is shared by the IL-4 and IL-13 receptors. It therefore acts on IgE class switching, eosinophil trafficking, FeNO, mucus secretion, and barrier dysfunction. Among available biologics, it has the broadest mechanistic connection to the SE-IL-4/IL-13-IgE-barrier circuit, but it does not directly neutralize SEs or Hla.

In patients with severe asthma and CRSwNP, dupilumab provides benefit in both compartments [63,64]. The fifth Bagnasco and Caruso study included here evaluated patients with severe allergic asthma who were nonresponsive to omalizumab and observed clinical improvements after switching to dupilumab [65]. This finding supports the value of changing the biological target when IgE neutralization alone is insufficient; in the absence of SE-sIgE stratification, it does not demonstrate that omalizumab nonresponse was caused by enterotoxins.

### 7.6. Tezepelumab: Upstream TSLP Blockade

Tezepelumab neutralizes TSLP, an epithelial alarmin linking barrier damage to dendritic cells, ILC2s, mast cells, and T2 responses. NAVIGATOR demonstrated reduced exacerbations across a broad spectrum of patients with severe asthma, with a greater absolute effect when eosinophils or FeNO were elevated [66]. Because Hla and SEB can damage the epithelium, TSLP blockade is conceptually attractive in a toxin/barrier phenotype. However, no published analyses are based on SE-sIgE, colonization, or local toxin (Table 3).

### 7.7. A Cautious Phenotyping Proposal

In patients with severe asthma, SE-sIgE may be considered an exploratory test when adult onset, eosinophilia, recurrent CRSwNP, elevated total IgE without clear aeroallergen sensitization, or persistent obstruction coexist. The result should be interpreted together with sinus endoscopy/CT when indicated, eosinophils, FeNO, total IgE, atopy, exacerbation frequency, and corticosteroid dependence. Nasal or sputum culture should be requested for a concrete microbiological question, not to “confirm” SE-sIgE.

Biologic selection should continue to be based on regulatory indications, validated biomarkers, comorbidities, age, preferences, and previous response [1,2,67,68]. Significant CRSwNP may favor dupilumab, mepolizumab, or omalizumab according to the overall profile; eosinophilia and steroid dependence support anti-IL-5/IL-5R therapy; elevated FeNO and IL-4/IL-13-related comorbidities support dupilumab; low or multiple biomarkers may orient treatment toward tezepelumab. SE-sIgE may strengthen the concept of a microbially amplified T2 endotype, but it does not select a drug on its own.

## 8. Knowledge Gaps and Research Agenda

The main knowledge gap is the distance between the serum biomarker and the mucosal source. Future studies should measure, at the same time, quantitative nasal and bronchial colonization; strain genome and transcriptome; local SE and Hla concentrations; serum and tissue SE-sIgE; TCR-Vbeta repertoire; cellular endotype; and barrier integrity. Longitudinal sampling before and after biologic therapy would clarify whether clinical improvement precedes or follows ecological changes.

Pragmatic trials or prospective studies with prespecified interaction between SE-sIgE and treatment are priorities. A biomarker-stratified design could compare responses to omalizumab, dupilumab, and anti-IL-5/IL-5R therapy in SE-sIgE-positive patients, avoiding indirect comparisons across cohorts. For antimicrobials, endpoints should include recolonization, resistome, and toxin production, not culture alone. For antitoxin strategies, patients with demonstrated active toxin should be selected: treating IgE memory alone would risk intervening on an immunological imprint no longer sustained by exposure.

Finally, three causality problems must be addressed: (i) reverse causality, whereby inflammation and corticosteroids favor colonization; (ii) confounding by CRSwNP, which may cause both greater exposure and more severe asthma; and (iii) toxin heterogeneity, because SEB-sIgE and SEA/SEC/TSST-1 mixtures are not interchangeable. Longitudinal methods, Mendelian randomization where possible, and experimental models using realistic mucosal exposures could reduce these uncertainties.

A cross-compartment research program should compare nasal, bronchial, skin, and intestinal isolates at strain level; quantify toxin transcripts and proteins; characterize biofilm and EV-associated cargo; and relate these measures to barrier function, SE-sIgE, and longitudinal asthma outcomes. Microbiome-directed trials should require documented carriage and include airway-specific biological endpoints, recolonization, resistome effects, and clinical outcomes.

## 9. Conclusions

*S. aureus* toxins offer a plausible paradigm of interaction among the microbiota, barrier function, and immunity in asthma. SEs act as superantigens and allergens, promoting T-cell activation, IgE, mast cells, and eosinophils; Hla and SEB may compromise the epithelium; and CRSwNP represents the main clinical and immunological reservoir of this circuit. Associations between SE-sIgE and severe asthma are convincing, but they do not amount to proof that the toxin is present or causally active in every patient.

Biologic therapies can interrupt different segments of the cascade: IgE, IL-5/IL-5R, IL-4/IL-13, or TSLP. A favorable response should not be described as eradication of *S. aureus* or toxin neutralization. At present, SE-sIgE is a promising research biomarker, particularly interesting in severe asthma with CRSwNP, but it is not a sufficient test for prescribing antibiotics or independently selecting a biologic. Clinical translation will require studies that connect, in the same patient and over time, bacterial strain, active toxin, immune response, and therapeutic outcomes. Evidence from intestinal ecology and AD expands the model by identifying extra-airway reservoirs, bidirectional barrier–microbe loops, biofilms, and EV-mediated toxin delivery. These mechanisms generate testable hypotheses but remain indirect for asthma and do not currently justify microbiome-directed treatment or decolonization in routine care.

## Figures and Tables

**Table 2:** Trials and finding about SEB and asthma.

Study	Design and Population	Main Finding	Interpretative Limitation
Barranco et al., 2021 [51]	Observational; asthma stratified by severity and atopy	SEB-sIgE associated with greater severity	Small sample size; causality cannot be assessed
Caruso et al., 2023 [12]	Italian multicenter retrospective study; 249 patients with severe asthma	25.3% SEB-sIgE-positive; association with CRS/CRSwNP, not with atopy or exacerbations	Single toxin marker; heterogeneous treatment
Won et al., 2023 [40]	Prospective cohort; 223 older adults with asthma and 89 controls, 2-year follow-up	SE-sIgE associated with persistent obstruction and exacerbations	Confounding by age, smoking, and disease duration
Schoini et al., 2024 [42]	50 patients with severe asthma; sputum, BAL, and biopsy	SE-sIgE associated with worse functional indices, not clearly with remodeling	Only 12 positive patients; many already receiving biologic therapy

**Table 3:** Biologics and the *S. aureus*-asthma circuit.

Biologic/Class	Blocked Node	Rationale in Relation to Toxins	What Cannot Be Claimed
Omalizumab	Free IgE/Fc-epsilon-RI	Potentially neutralizes SE-sIgE and IgE-mediated degranulation [57].	Does not block superantigenicity, colonization, or Hla
Mepolizumab/reslizumab	IL-5	Reduces eosinophil survival fueled by Th2/ILC2 pathways [58,61].	No evidence of an effect on bacterial burden or toxin
Benralizumab	IL-5Ralpha	Depletes eosinophils, central effectors of the SE-sIgE/CRSwNP phenotype [59,60,62].	Not an antitoxin therapy; residual neutrophilic circuits may persist
Dupilumab	IL-4Ralpha: IL-4 and IL-13	Reduces IgE class switching, FeNO, mucus, and barrier dysfunction [63,64,65].	Does not neutralize SEs/Hla; superiority in SE-sIgE-positive patients has not been tested
Tezepelumab	TSLP	Intercepts the upstream epithelial signal after microbial injury [66,67].	No trial stratified by *S. aureus* or SE-sIgE

## Data Availability

No new data were created or analyzed in this study.
